# The role of the smartphone in the transition from medical student to foundation trainee: a qualitative interview and focus group study

**DOI:** 10.1186/s12909-018-1279-y

**Published:** 2018-07-31

**Authors:** John E. A. Shenouda, Bethany S. Davies, Inam Haq

**Affiliations:** 1 Division of Medical Education, Brighton and Sussex Medical School, Room 344A, Mayfield House, University of Brighton, Falmer, Brighton, BN1 9PH England; 2 Sydney Medical Program, Rm 208, A27 – Edward Ford Building, The University of Sydney, Sydney, NSW 2006 Australia; 3 Department of Global Health and Infection, Brighton and Sussex Medical School Teaching Building, University of Sussex, Brighton, East Sussex BN1 9PX England

**Keywords:** Institutions, Junior doctor, Medical student, Smartphone, Transition

## Abstract

**Background:**

The transition from medical student to junior doctor is one of the most challenging in medicine, affecting both doctor and patient health. Opportunities to support this transition have arisen from advances in mobile technology and increased smartphone ownership.

**Methods:**

This qualitative study consisted of six in-depth interviews and two focus groups with Foundation Year 1 Trainees (intern doctors) and final year medical students within the same NHS Trust. A convenience sample of 14 participants was recruited using chain sampling. Interviews and focus groups were recorded, transcribed verbatim, analysed in accordance with thematic analysis and presented below in keeping with the standards for reporting qualitative research.

**Results:**

Participants represented both high and low intensity users. They used their smartphones to support their prescribing practices, especially antimicrobials through the MicroGuide™ app. Instant messaging, via WhatsApp, contributed to the existing bleep system, allowing coordination of both work and learning opportunities across place and time. Clinical photographs were recognised as being against regulations but there had still been occasions of use despite this. Concerns about public and colleague perceptions were important to both students and doctors, with participants describing various tactics employed to successfully integrate phone use into their practices.

**Conclusion:**

This study suggests that both final year medical students and foundation trainees use smartphones in everyday practice. Medical schools and healthcare institutions should seek to integrate such use into core curricula/training to enable safe and effective use and further ease the transition to foundation training. We recommend juniors are reminded of the potential risks to patient confidentiality associated with smartphone use.

## Background

Medical transitions are those in which doctors or medical students move from one significant stage in their training to the next. Examples include starting as a newly qualified junior doctor or taking up a consultancy post. These transitions have been described as “critically intense learning periods” in which doctors continue to learn, but with an immediate expectation to deliver optimal results upon initiating new jobs [1].

The transition from medical student to junior doctor is recognised as being highly stressful [2, 3]. Particular challenges include frequent rotation around new working environments, new responsibilities and the challenge of inexperience. Such stress may result in a reduced quality of patient care through leading to inefficient care activities or poor communication between colleagues, as a result of reduced emotional well-being [4]. Despite the greater level of responsibility placed upon final year students, junior doctors still feel unprepared when it comes to decision making, prescribing, roles within the medical hierarchy and performing under stress [5–7]. This clinical uncertainty can translate into poor patient and treatment outcomes highlighting the importance of helping foundation trainees through this transition [4, 8].

The rise in ownership of mobile technology among medical students [9, 10] has provided a platform upon which transitions could be aided through the use of mobile clinical resources and multiple modes of communication [10, 11]. As such, medical educators have looked to implement mobile devices within learning and teaching interventions, with interest currently centring on Smartphones [12–14]. Mobile technology allows for just-in-time learning, learning in context and can support decision making [10, 15, 16]. Smartphones offer increasingly faster computing, internet access, high quality cameras as well as communication capabilities. The literature to date on the outcomes of smartphone-based educational interventions is still relatively limited, but evidence is growing, such as that from the iDoc project [16, 17]. More specifically, Dimond et al. in their work suggested that smartphones can support the process of transition by providing rapid access to information [18]. However, their work looked at one specific app, rather than looking more broadly at smartphone use as a whole, in all its capacities.

A further consideration is that disruptive technologies such as these encounter a number of barriers to clinical utilisation, including confidentiality and security concerns [10, 15, 19]. Earlier work by Davies et al. suggested that a change in attitude, behaviour and approach is required by both teacher and learner; and that both individual and institutional input is required to maximise opportunities [15]. Additionally, performance in transitions is not merely the responsibility of the doctor, but of the medical school and workplace [1, 20].

Educational research around transition has contributed to the restructuring of undergraduate training and the Foundation Programme (the first two years working as a clinician after graduating with a primary medical qualification) [21, 22]. Therefore, this study sought to further explore the role of the smartphone for final year medical students and foundation trainee doctors in an attempt to appreciate how and why they are using their smartphones for work and for learning, especially during the process of ‘responsibility transition’; and what issues, if any, require external support or intervention.

## Methods

### Ethics, consent and permissions

Ethical approval for the study was given by the medical school Research Governance and Ethics Committee (14/033/HAQ). Informed consent to participate was obtained (as described below) from all participants, as well as consent for the publication of their quotations.

### Participants and recruitment

**Fig. 1:**
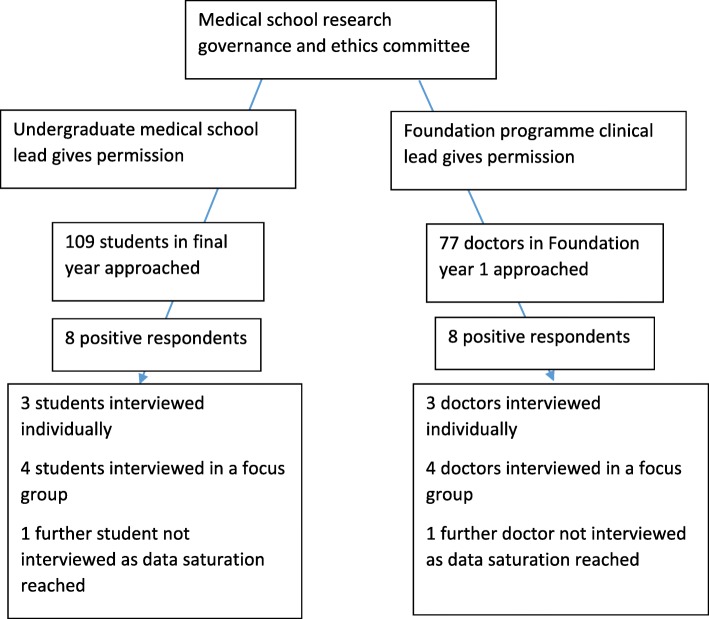
A flowchart to show the process leading up to data collection

The study was advertised through emails, weekly bulletins, announcements and flyers. Inclusion criteria for recruitment were being a Foundation Year 1 doctor or a final year medical student based at the study site. There were no specific exclusion criteria. Participants were not remunerated in order to avoid recruitment bias. Fig. 1 is a flow diagram which depicts the process. Sixteen potential participants responded from a cohort of 77 Foundation doctors and 109 medical students (overall response rate 8.6%). This was a convenience sample, and by getting participants to recommend friends, we also used chain (or snowball) sampling. Fourteen participants were divided into two focus groups of four and six individual semi-structured interviews. Two further candidates were not interviewed as data saturation, described below, was reached.

### Methodology

This study used focus groups and interviews to examine smartphone use in the transition to foundation training. An interview schedule was devised on the basis of an initial literature review and following discussion between the authors. The schedule was refined over the course of the interviewing period to explore emerging themes from existing data. The decision to include both individual face-to-face interviews and focus groups was taken on a pragmatic basis, as each method brings different advantages and disadvantages; and also allowed for participant availability and preference.

Informed signed consent was obtained before every interview and focus group. All interviews were performed by the same researcher (JS) to ensure a standardized procedure and took place at mutually agreed locations. Data collection took place over a period of two months. Audiotape recordings were made, transcribed verbatim professionally, and then checked for accuracy by JS and BD. All audio recordings were labelled anonymously. Thematic analysis was used to analyse the data, employing within and cross case analysis to develop themes. Cross-referencing was used to search for patterns separately across the student and doctor groups. Disconfirming evidence was also sought. The number of interviews conducted was determined by data saturation on the major themes, identified when no new data emerged from the semi-structured interviews in either cohort. JS and BD independently coded and analysed the transcripts, then compared and integrated the themes through discussion together with IH. The manuscript was subsequently compiled by JS, BD and IH in accordance with the Standards for Reporting Qualitative Research (SRQR) guidelines [23].

## Results

Interviews ranged from 25 min to 37 min in length with the focus groups both lasting roughly 50 min (see Table 1). Quotations presented as results are labelled with the participant role, study number and gender (eg. FY1, 11F meaning from the Foundation Year 1 cohort, participant number 11 who is female). Participants’ ages were between 22 and 31 years.

**Table 1:** Participant interview data

Participant number and gender	Foundation Year 1 Trainee (FY1) or Final Year Medical Student (MS)	Focus group or Interview	Duration of interview (minutes)
1M	MS	Focus Group	48 m:55 s
2M	MS	Focus Group	
3F	MS	Focus Group	
4M	MS	Focus Group	
5F	MS	Interview	30 m:33 s
6M	MS	Interview	36 m:03 s
7F	MS	Interview	27 m:16 s
8M	FY1	Focus Group	51 m:51 s
9F	FY1	Focus Group	
10M	FY1	Focus Group	
11F	FY1	Focus Group	
12F	FY1	Interview	24 m:10s
13F	FY1	Interview	33 m:21 s
14M	FY1	Interview	33 m:04 s

As well as including both final year students (MS) and Foundation Year One trainees (FY1), the participants represented a variety of low and high intensity phone users:

“I do think I use my phone a lot less than other people do. […] I don’t really know why. I’m just not very good with phones.” (MS, 12F).

“I found myself probably slightly at the other end of the scale to [1M], where I would say I use my smart phone every time I actually go into hospital for something.” (MS, 2M).

The commonest themes identified from the data were the use of MicroGuide™, instant messaging, photographs and concerns about perceptions of use by patients and colleagues. The fifth theme (differences between FY1 and MS) was deliberately sought.

### MicroGuide™

Smartphones were used by participants to support their prescribing, especially with regards to antimicrobial agents. Ten of the 14 participants specifically brought up their use of the MicroGuide™ app without prompting by the researcher. This is a smartphone application which allows clinicians to access local antimicrobial guidelines for a specific hospital trust. One participant described it as a “lifesaver” (FY1, 8M) and for another, it constituted their primary use of their phone for work:

“I think the only real time I use my smart phone is looking at looking antibiotic guidelines in the hospital.” (MS, 7F).

Key aspects that contributed to use of MicroGuide™ were: that it only required intermittent internet access (4M, 11F); that it was more convenient than trying to get onto a ward desktop computer (7F, 8M, 13F, 14M) or using a paper formulary (2M, 14M); and that it was trust-specific:

“Trust-specific apps are really good, like the MicroGuide one, I don’t think there was anything like that in Wales, where I came from, and that’s so useful to have something that specific, because anti-microbial guidelines are really specific to trusts.” (FY1, 13F).

“I do actually have the MicroGuide app that I downloaded whilst I was at the other regional centre, because they had different prescribing guidelines.” (MS, 1M).

Another positive aspect was that they found it to be user friendly, organised in a way that fitted with clinical practice (2M, 13F, 14M):

“It’s convenient, it’s in your pocket, you could be anywhere in the hospital, you pull it out, open the app, it’s organised by body system, so you can just go in, you know what you’re looking for and immediately you find it, rather than […] going onto a ward, looking for the BNF [British National Formulary], then scrolling […] through the index to find the letter of the drug that you want, then going to that page. It’s a lot quicker, it’s a lot more efficient.” (FY1, 14M).

“It’s for antibiotic guidelines and specific to this Trust. So you just click on the body system and then […] whether it’s a severe infection or not, and it gives you the dose and type of antibiotic and duration. It’s really useful.” (FY1, 13F).

The disadvantages that had arisen were related to the acceptability of smartphone use during clinical encounters:

“As an F1 [FY1] you do, often on a ward round, the consultant will say “Oh, start antibiotics for HAP [hospital acquired pneumonia]” or something, and you’re like “Oh, what was that dose again?,” so you quickly look it up, and then you wonder, does the consultant think that I’m just playing on my phone, and does the patient think that I’m just playing on my phone? That’s the only downside. You don’t really have time to like pop out, do it in private and then come back again, when you’re on a busy ward round.” (FY1, 13F).

“So you could be looking up, like (for example), the guidelines for antibiotics for a chest infection, but the patients don’t know that that’s what you’re doing, and I just think it doesn’t look particularly good.” (FY1, 12F).

### Perceptions of use

The issue of appearing to use mobiles for non-clinical purposes was not solely related to the MicroGuide™ app, but was a general problem that had been encountered by participants, both students and FY1s, as 12 of the 14 discussed:

“I think there [are] still some people that can be offended by it, and especially as a medical student, if you’re fiddling around on your phone, even just to look something up, or to do something relevant and useful, people might still think you’re texting someone. Because it’s just what it always looks like when you’ve got your phone, it looks like you’re texting someone, and I think that until we get over that, I’m always going to feel slightly uncomfortable using my phone on the wards.” (MS, 4M).

“I think sometimes when you whip out [take out] your phone in the hospital, people assume that you’re using it for personal reasons, not to look things up.” (MS, 7F).

“If you’re checking your phone, or, even if you’re not speaking to them directly, you’re just on the ward, or at the desk; if they see that, and it’s their opinion that you’re texting your mate [friend] or doing something else, then they may perceive that as unprofessional.” (MS, 2M).

Five participants had experienced occasions where a patient or clinician had voiced their disapproval of smartphone use (5F, 6M, 8M, 11F, 12F):

“I’ve been in clinic, as a student, with somebody else who was looking something up on their phone, and the patient actually said something to the doctor about it, and it was really uncomfortable.” (FY1, 12F).

“I’ve had one consultant, not to me, but to someone else on my firm, and say “What are you doing with that phone? Can I just check that you are doing something […] related to why you’re here”, rather than assuming that he was texting, but he did ask, he didn’t just assume. But still, he noticed that this guy [person] had his [phone out]...” (FY1, 11F).

However, for one individual, it was appropriately so, having been found to be playing a game during an outpatient clinic.

As a result of these perceptions, participants had developed various ways of successfully incorporating their smartphone use into their clinical practice. Ten of the participants volunteered their own personal tactics:

“I kept telling her “I’m just looking that up” when I got my phone out, to make sure she knew, because I wouldn’t want to [...] look rude.” (MS, 5F).

“I think we have to be careful, you shouldn’t just pull your phone out in front of patients and stand as though you’re texting or... you need to be careful and selective when you do decide to take your phone out. However, I do think there’s times where sometimes you need your phone out in the middle of the ward and you just want to quickly check something and I think that’s fine to do so.” (FY1, 14M).

“I have used my phone in front of patients, but I try to like make it look like I’m not using my phone.” (FY1, 12F).

“I won’t do it by the patient bedside, and I won’t do it in the middle of a ward round, unless you’re in-between patients. But I will quite comfortably stand at the desk and look up something on my phone, or if I’m filling out a drug chart, I have my phone next to me, and just be checking things about those drugs, while I’m going along.” (MS, 2M).

“The only time I ever use it in front of a patient is literally just for looking up doses, and then […] I vaguely hide it behind my clipboard.” (FY1, 9F).

### Instant messaging

Nine participants discussed their use of instant messaging (IM) via WhatsApp for clinically-related communications without prompting by the researcher; another two did so after specific prompting. The overarching purpose was for coordination of the work of the clinical team, increasing efficiency:

“Can you go see bed 6.2 and review the calcium […], and you can’t really do that as many times a day if you’re bleeping people, because you have to wait by a phone.” (FY1, 10M).

“Because everyone gets split up in different directions, especially on surgical ward rounds, so it’s really helpful to be like “Where are you?” and […] somebody will reply. Or to say, “I’ve done this job,” so that nobody else then has to go round to that end of the hospital to do it, or, if you’re around somewhere, you can ask someone if there’s anything else that wants doing over there.” (FY1, 12F).

Students also used IM for coordinating teaching sessions and learning opportunities (5F, 7F):

“When we’re in ____, the 13 of us would have just the same group where we […] mainly told each other where teaching was, or when teaching was.” (MS, 5F).

The asynchronous communication afforded by IM was key for six participants:

“The problem with the bleep system is [that] you have to answer your bleep straight away, because you don’t know what it is. It could be an emergency and you need to answer it. Whereas you can leave a message on the WhatsApp group for someone to do, and do it when they’ve got a free time to do it. Obviously I wouldn’t put a message saying, “Oh by the way, lady in Bed 9’s having a heart attack. Could someone go over there.” But for […] the routine jobs it’s quite useful.” (FY1, 12F).

“Quite often you want to pass a message to someone, but you don’t need them to do it right here, right now. You don’t want to shriek in their ear with that annoying sound that just haunts you.” (FY1, 9F).

However, this also brought its own disadvantages (3F, 7F, 12F, 13F, 14M):

“But then the other problem with that is then if you are trying to get hold of somebody, they don’t always answer their phone, or answer the message you send, whereas the likelihood is, they will answer their bleep.” (FY1, 12F).

This also led to issues when messages would arrive out of working hours (5F, 12F, 13F, 14M):

“Another bad thing is that if you do go home and you’ve still got your phone, […] you can leave [your bleep] with the next F1, but your phone you have to take home with you and [...] that’s not a very good work/life balance.” (MS, 5F).

Another factor with using IM was the issue raised by 6 participants of the need to maintain patient confidentiality when using IM:

“You wouldn’t be able to text “This patient needs seeing, they’re here and their name’s this,” because that’s obviously is a bit... that’s not as safe as emailing those details.” (MS, 5F).

“It’s such an easy way to communicate to people, obviously keeping it confidential.” (MS, 8M).

“We always only use hospital numbers, and never any kind of clinical information really, just “This patient is coming to Level 9, can you look out for them”.” (FY1, 13F).

### Photos

A final issue that arose as an important matter for participants was the use of smartphones to take clinical pictures. Nine of the participants discussed it without prompting, and another two after being asked. Most were clear that they would not do so, “I would never”, “I don’t think it’s professional”, “no, absolutely not”, “I would not take a picture, not take a picture of a patient”, “you’re not supposed to”. However, one of the 14 participants had actually done so, having been asked specifically by the consultant to do so:

“I have used it to take pictures, but you do have to be really careful to make sure that you don’t get anything that could identify the patient and you delete it straight away. I wouldn’t have done that normally, but my consultant asked me to, so he could [have] a photo of it. But I didn’t feel particularly comfortable having a picture of a patient on my phone. I’m not really sure if that’s allowed.” (FY1, 12F).

Two further had witnessed senior clinicians take photos:

“I’ve only seen them [more senior clinicians] use it for […] maybe sometimes sharing a photo with, like, to get someone’s opinion, but with the patient’s consent obviously.” (MS, 7F).

“I’ve seen consultants take photos.” (MS, 5F).

### Differences between MS and FY1

The main differences discussed by the participants between medical students and foundation doctors fell into three main subthemes. Students used their phones for learning; junior doctors used them in their clinical practice, and the feeling was that this was due to the demands of their work.

“I think as a student […] with all the (clinical) scores […], it’s more important to know what they’re for, rather than actually using them. Whereas as an F1, you’re actually using your phone to calculate something.” (FY1, 11F).

“As a student it was more working out what certain drugs were and what they did; whereas now you need to be certain of doses, or whether there’s any interactions or side effects.” (FY1, 13F).

“When you start work you need it specifically for certain uses that you haven’t used in the transition” (FY1, 14M).

They felt as if junior doctors had less time available to them within the working day;

“I don’t have any more games on my phone… I just […] deleted them, there was no time […]. There’s not many clinics and ward rounds that you do at the back, that you can play. […] Everything that you do, you have to pay attention […] now. There’s not much free time.” (FY1, 8M).

“I tend to find that I spend more time […] carrying notes and finding patient folders and doing all the stuff that goes with the ward round; rather than as a medical student, I used to look things up on my phone or actually use my phone to work out what on earth was going on.” (FY1, 11F).

“I’ve never personally even come close to running out of battery because I don’t have the time to use it that much.” (FY1, 9F).

However, they recognised the contribution to learning that phones offered:

“I’m looking stuff up on our Doctor Companion app all the time, so you’re just […] learning things from it in that way, that’s helpful, that’ll help me become an F1” (MS, 5F).

“I think I used my phone as a, like a learning tool a lot more as a medical student than as an F1.” (FY1, 11F).

Three junior doctors had found a way to still benefit from the time available to students:

“I get the medical students to look things up for me. So, if I don’t know what the patient’s got […], I vaguely fake it as […]homework and ask them to look it up, “Oh look that up” and then I find out.” (FY1, 9F).

“It’s a very interesting disease, you should learn about it.” (FY1, 10M) “And tell me as well.”(FY1, 8M)

## Discussion

The study reports that participants found smartphones to be a valuable resource in their clinical practice, aiding in the “responsibility transition” [24] from medical student to doctor. Smartphones were a tool for safe, appropriate prescribing and for coordinating clinical work. They offered alternative means of communication which complemented the bleep system, especially through instant messaging. The disapproval experienced with clinical smartphone use and unprofessional behaviour were identified as the greatest limiting factors affecting use. As is well documented in the literature [9–12], the professional aspects of clinical smartphone use are a problem, and influenced the use of smartphones by our participants. The analysis also adds to the body of knowledge raised by Dimond et al., around the rituals associated with mobile phone use in the clinical setting [18].

Other findings which are similar to those found elsewhere, include an acknowledgement that smartphones support student learning, time management and communication with peers, teams and the medical school [25]. Doctors stressed that smartphone apps were not essential to practising safely but saved time [13, 26] and ensured that local guidelines were adhered to. Unsurprisingly, the differences in smartphone use between students and doctors reflected their differing needs: learning and working respectively, corroborating literature in the field [9, 27].

### Contribution to the literature

There are 3 key contributions of this study to the literature on smartphones in transition. Firstly, the role of trust-specific antimicrobial prescribing apps; secondly, the use of instant messaging by doctors; and finally, the practice of taking patient photographs with a smartphone.

### Trust-specific antimicrobial prescribing apps

Monrouxe et al. have recently shown that UK medical graduates are underprepared for certain aspects of clinical practice [7]. They identified core skills including prescribing and clinical reasoning/diagnoses among others. They concluded that educational interventions were needed to address these areas. Previous work by the same group had demonstrated that access to an app-based textbook had provided valuable support to newly qualified doctors during a period of transition [16, 17]. We argue that our findings suggest that prescribing support, such as that provided through the MicroGuide™ app, are of great value to those transitioning from final year student to practising junior doctor, and could help improve graduate preparedness for the workplace. This is of further importance when considering that greater than 50% of new graduates move to different training regions from where their undergraduate medical teaching took place [28] and that the majority of prescribing in secondary care is undertaken by junior doctors [29]. As called for by Ryan et al. [30], our findings suggest that one such intervention to help reduce medication error among FY1s and improve patient safety during the transition could be widespread availability of such an app.

In addition, these are timely findings to help inform the current problem for antimicrobial stewardship. The crisis of antibiotic resistance poses a global threat for which many different stakeholders are responsible; including individual healthcare professionals [31]. Our research suggests that users found trust-specific antimicrobial applications advantageous in their clinical practice, aiming to ensure safe and appropriate prescribing. Easily accessible guidelines were used to reduce prescribing errors, and improved adherence to local policy. Such a step, though seemingly small, may ease the responsibility transition as a newly qualified doctor and additionally aid working towards antimicrobial stewardship as set out in the 2016/17 national CQUIN goals [32].

### Instant messaging

This study showed that smartphones were used together with the paging system, rather than replacing it. This was through the use of instant messaging (IM) rather than by telephone calls. Instant messaging is a form of (mostly) text based communication between participants over the internet, which appears to occur in real time. This is not yet widely described within the literature [33], but is an important example of how juniors are working. IM offered benefits (like Raiman et al.) in facilitating communication to coordinate work duties and teaching [33]. Clinical uncertainty due to inexperience, is most frequent among newly qualified doctors for which having real time access to peers or near-peers, such as Foundation Year 2 or Core Trainee doctors, can help the transition by reaffirming clinical judgement and reducing clinical error, ultimately improving patient safety. This is of paramount importance given the associated risk to patient safety with the start of newly qualified doctors [34, 35]. Having multiple means of communication eased clinical activities for junior doctors despite unintended consequences [11, 36–38], and our study shows that this holds true for IM too.

One negative aspect of IM was an intrusion into participants’ life outside of work, an “empowerment-enslavement” paradox [39]. As Jarvenpaa et al. described, the advantages of constant availability are weighed against the disadvantages. It is worth noting that participants believed the benefits to patient care and professional development outweighed the annoyance of constant availability. Additionally, concerns over confidentiality were raised, as participants were aware that patient identifiers should not be used. Those who were using IM used WhatsApp rather than a secure IM app designed for hospital use; WhatsApp does now have end-to-end encryption, but did not at the time of the study. These issues surrounding the use of instant messaging need further study – how widespread is the practice, how are the juniors regulating their behaviour, is it safe for patients? Identifying these could offer opportunities to instigate change that improve practice within the hospital, especially where IM use is widespread. Furthermore, such change could positively integrate the use of secure instant messaging apps, many of which are free to use, into clinical practice, the importance of which continues with seniority in the medical field.

### Smartphone photography

In this study, both students and doctors were concerned about the use of clinical smartphone photography, as they believed that patient confidentiality may be at risk. This accords with NHS and local policy. However, a small but relevant few reported either taking photographs on their own phone, or witnessing others doing so. The rationale is understandable – they were encouraged by senior colleagues, and they could see an instant benefit to patient care. This is similar to others’ findings, such as FY1s using photographs to help in clinical decision making with regard to patient care [40]. However any clinical photography on a smartphone directly contravenes current local hospital policy. This is an important finding in the context of transition whereby newly qualified doctors are left balancing obedience to the medical hierarchy versus adhering to local policy. In turn, the concept of challenging the medical hierarchy, who rightly boast vast clinical experience over new graduates, may result in them feeling inferior or unable to question from an educational and patient safety standpoint: “I thought you knew best and I shouldn’t interfere.” [41].

Given the medico-legal risks of clinical photography [42] and doctors’ lack of awareness of the legal considerations [43, 44], we recommend that students and junior doctors should avoid the use of clinical smartphone photography at present. What concerns us more is the fact that the three reported episodes were all consultant-led. According to Lave and Wenger, learners acquire beliefs and behaviours through their involvement in a community of practice [45]. A culture which accepts and promotes such practices is likely to result in such behaviour being perpetuated by junior clinicians despite the risks. By highlighting this issue within this study, we hope that it raises awareness and confidence amongst junior clinical staff to consider their own practices with photography. However, it would be helpful to explore how best to use smartphone photography for patient care while minimising the risks. There appears to be a mismatch between practice and policy currently, so other interventions may be required. Smartphones are an example of “disruptive technology”, i.e. one that disturbs or threatens established practice but which holds potential to transform care opportunities [46, 47]. However, as with the plethora of other medical technologies introduced every year, the health sector remains slow to respond [19] despite potential clinical and patient benefits [48]. It may be that experience garnered from other technologies such as telehealth could offer a potential framework by which to incorporate it into health systems [49]. Another alternative is to provide a different solution, such as investing in software that would allow clinicians to take mobile photographs in a secure way [50].

### The hidden curriculum

Smartphone use was a skill to master, with participants developing various ways in which they were able to successfully integrate phone use into their practice, consistent with the behaviours described by others [18, 51]. This may be a manifestation of the ‘hidden curriculum’ [51, 52] in which students and doctors learn the art of using smartphones outwith their core educational curricula. This is important to consider and reflect on [53]. As Wear describes, “As a vast network of unwritten social and cultural values, rules, assumptions, and expectations, the hidden curriculum shapes behaviour so much that mastery of the hidden curriculum is as important as mastery of the formal one.” [53]. Students, doctors and patients could benefit if this were recognised and formalised, just as communication skills moved from being a hidden curriculum to being a foundation of core curricula essential for good patient-centred care [54, 55]. This may already be the case in some centres [55], but is not yet widespread. It could be achieved, for example, by inclusion within the GMC’s *Tomorrow’s Doctors* [56], much as it currently includes a requirement for medical schools to “take advantage of new technologies to deliver teaching”.

Smartphones will not be useful for all, and their clinical use has shortfalls, as we, and others, have described. They “are not intrinsically good or bad, desired or rejected” but need to be understood in the context in which they are used [51]. Honest and open discussion must be encouraged to direct the role of smartphone technology in healthcare. In the meantime, we would recommend that induction and placement shadowing include the issue of how best to use smartphones in the workplace. Taking the findings both from our study, and others [57, 58], we suggest trainees be specifically reminded of the risk of breaching confidentiality through common smartphone uses such as instant messaging, photographs and even phone conversations.

### Limitations and future research:

This study has a number of limitations. The small size of the study and its single sited nature could raise concerns about how robust the findings are. However, we reached data saturation on the themes, and had further potential participants that we could have used if we had deemed it necessary. The agreement of some of our findings with those of other research groups is also reassuring, such as the issues around perception and rituals of use.

Another limitation of the study was the low response rate of recruitment. We are unsure why the rate was low. Possibilities include participant ‘fatigue’ – there are a number of research projects involving medical student participants, and only a relatively small cohort of students to recruit from. Another is whether people self-excluded themselves due to the topic of smartphone use, through lack of interest or feeling as if they would not be able to contribute. This may also have contributed to selection bias in the form of recruiting positive respondents, all of who were smartphone owners, interested enough in the topic to join the study. However, participants varied in the extent to which they used their phones. We felt that we had a spectrum of ‘users’ versus ‘non-users’ giving a fair reflection of the final and foundation year cohorts.

The methods used mean that the data was all self-reported by participants. We did not have the resources in place to be able to conduct observational field work for this study, which would provide an alternative viewpoint and could be the focus of future research adopting a phenomenological approach. However, the methods are similar to many other studies within the field. We have also tried to limit the effect of researcher bias, both through a process of reflexivity, as well as open discussion between ourselves; and looking specifically within the data for disconfirming evidence to check or amend the conclusions we made.

A final consideration is the speed at which the technology moves on, and the inevitable delay between data collection and publication. However, we feel that the issues we have raised remain relevant to current junior clinicians.

## Conclusion

In this sample, both final year medical students and foundation trainees used smartphones in their everyday practice. The evidence within the literature lags behind what is happening in hospitals and medical schools. This study contributes to this knowledge, especially with regard to the process of transition from final year student to junior doctor. They used their smartphones to support their prescribing practices, especially antimicrobials. Instant messaging contributed to the existing bleep system, allowing coordination of both work and learning opportunities across place and time. Clinical photographs were recognised as being against regulations but there had still been occasions of use despite this. Concerns about public and colleague perceptions were important to both students and doctors, with participants describing various tactics employed to successfully integrate phone use into their practices.

We would recommend that hospitals and medical schools support them in this process by bringing this hidden curriculum into core curricula. This may allow benefits to be harnessed, while limiting disadvantages. Specific recommendations would include:

1. Training – including confidentiality issues and clinical photography
2. Trust specific apps, such as the antimicrobial guidelines
3. Secure messaging service such as http://medxnote.com/
4. Secure clinical photography service such as http://picsafe.com/medi

Likewise, a reminder to senior colleagues with regards to the current do’s and don’ts of clinical photography would seem timely.

## Abbreviations

BNF: British National Formulary
CQUIN: Commissioning for Quality and Innovation
FY1: Foundation Year 1 doctor
GMC: General Medical Council
IM: instant messaging
MS: medical student
NHS: National Health Service
PDA: personal digital assistant
SRQR: Standards for Reporting Qualitative Research
